# Radial BMD and serum CTX-I can predict the progression of carotid plaque in rheumatoid arthritis: a 3-year prospective cohort study

**DOI:** 10.1186/s13075-021-02642-4

**Published:** 2021-10-13

**Authors:** Seungwoo Han, Na-Ri Kim, Jong-Wan Kang, Jung-Su Eun, Young-Mo Kang

**Affiliations:** 1 Division of Rheumatology, Department of Internal Medicine, Kyungpook National University, School of Medicine, 680 Gukchaebosang-ro, Junggu, Daegu, 41944 Republic of Korea; 2 Division of Rheumatology, Department of Internal medicine, Daegu Fatima Hospital, Daegu, Republic of Korea

**Keywords:** Rheumatoid arthritis, Atherosclerosis, Risk factor, Bone mineral density, C-terminal telopeptide of type-I collagen

## Abstract

**Objective:**

Patients with rheumatoid arthritis (RA) are almost twice as likely to develop cardiovascular disease (CVD) as those without. However, traditional CVD risks have been shown to underperform in RA patients; thus, we aimed to identify new surrogate risk factors to better reflect their atherosclerotic burden.

**Methods:**

A total of 380 RA patients with carotid atherosclerosis data were analyzed in this prospective cohort study. The primary outcome was carotid plaque progression over the 3-year follow-up period. Risk parameters assessed for the progression of carotid plaque were categorized as demographics, traditional CVD risks, RA-related risks, and bone parameters.

**Results:**

The progression of carotid plaque was associated with the level of rheumatoid factor (*p* = 0.025), serum C-terminal telopeptide of type-I collagen (CTX-I) (*p* = 0.014), and femur and distal radius bone mass density (BMD) (*p* = 0.007 and 0.004, respectively), as well as traditional CVD risk factors. In multivariable analyses, the bone parameters of serum CTX-I and distal radius BMD proved to be independent predictors of the progression of carotid plaque along with hyperlipidemia, smoking, and baseline carotid plaque (all, *p* < 0.05). Adding both serum CTX-I and distal radius BMD increased the carotid plaque progression prediction model’s percentage of explained variance from 24 to 30%.

**Conclusion:**

High serum CTX-I and lower radius BMD, reflecting high bone turnover, were independent risk factors for the progression of carotid plaque in RA patients, implicating the direct or indirect role of bone metabolism on the atherosclerotic burden.

**Supplementary Information:**

The online version contains supplementary material available at 10.1186/s13075-021-02642-4.

## Introduction

Patients with rheumatoid arthritis (RA) are nearly twice as likely to die before the age of 75 compared with people without the disease. The leading cause of this excess mortality among all age groups is cardiovascular disease (CVD) [1]. This is mainly mediated by the increased burden of atherosclerosis in RA patients. However, identifying RA patients at risk for CVD is challenging since the standard CVD risk stratification tools developed for the general population have been shown to underperform in RA patients [2]. Indeed, the Framingham risk equation for predicting CVD outcomes failed to predict almost half of the events that would occur in RA patients [2]. Thus, attempts have been made to establish a RA-specific CVD risk prediction equation [3, 4], but external validation has failed to uniformly predict the CVD risk in RA populations [4]. Moreover, traditional risk factors justify only about 20% of the variance in the total burden of subclinical atherosclerosis, even in the general population [5]. This highlights the need for a new risk surrogate that better reflects the atherosclerotic burden, especially in RA patients.

The cellular component of atherosclerotic lesions predominately comprises smooth muscle cells and macrophages [6]. Macrophages surrounded by calcium deposits can have an anti-inflammatory phenotype of the M2 macrophage, which could potentially display osteoclast (OC)-like phenotype [7]. Actually, tartrate-resistant acid phosphatase (TRAP)-positive OC-like cells have been identified in atherosclerotic lesions, and the knockout of cathepsin K, the OC-specific lysosomal protease, shows an attenuated atherosclerotic progression, suggesting that OCs play a role in the pathogenesis of atherosclerosis [8, 9]. In addition, atherosclerotic lesions have osteoblast-like cells, which are transdifferentiated from vascular endothelial cells, smooth muscle cells, and mesenchymal cell-like fibroblasts [10]. It is well known that the osteoclast and osteoblast involving bone remodeling play a critical role in the pathogenesis of atherosclerosis, which is mainly induced by inflammation followed by vascular wall mineralization [11]. Along with these basic and translational studies, recent clinical reports also suggest that decreased bone mineral density (BMD) is an independent predictor for CVD [12].

To investigate the risk factors for CVD and its contribution to mortality in RA patients, we established the Kyungpook National University Hospital (KNUH) Atherosclerosis Risk in RA (KARRA) cohort. In the previous study,  we observed that the presence of carotid plaque in RA patients was associated with a cumulative inflammatory burden, such as the cumulative erythrocyte sedimentation rate (ESR) and health assessment questionnaire (HAQ) score, as well as current disease activity, including the Disease Activity Score-28 using ESR (DAS28-ESR) [13]. However, those results were drawn-out under cross-sectional design during the period of cohort enrollment; consequently, it was hard to conclude the causal and temporal relationships between the risks and carotid plaque. The aim of this 3-year prospective, longitudinal cohort study was identification of a novel surrogate risk factor to predict the progression of subclinical atherosclerosis in RA patients and, in particular, to evaluate whether bone-related variables are associated with the atherosclerosis burden.

## Patients and methods

### Study design and patients: the KARRA cohort

**Fig. 1:**
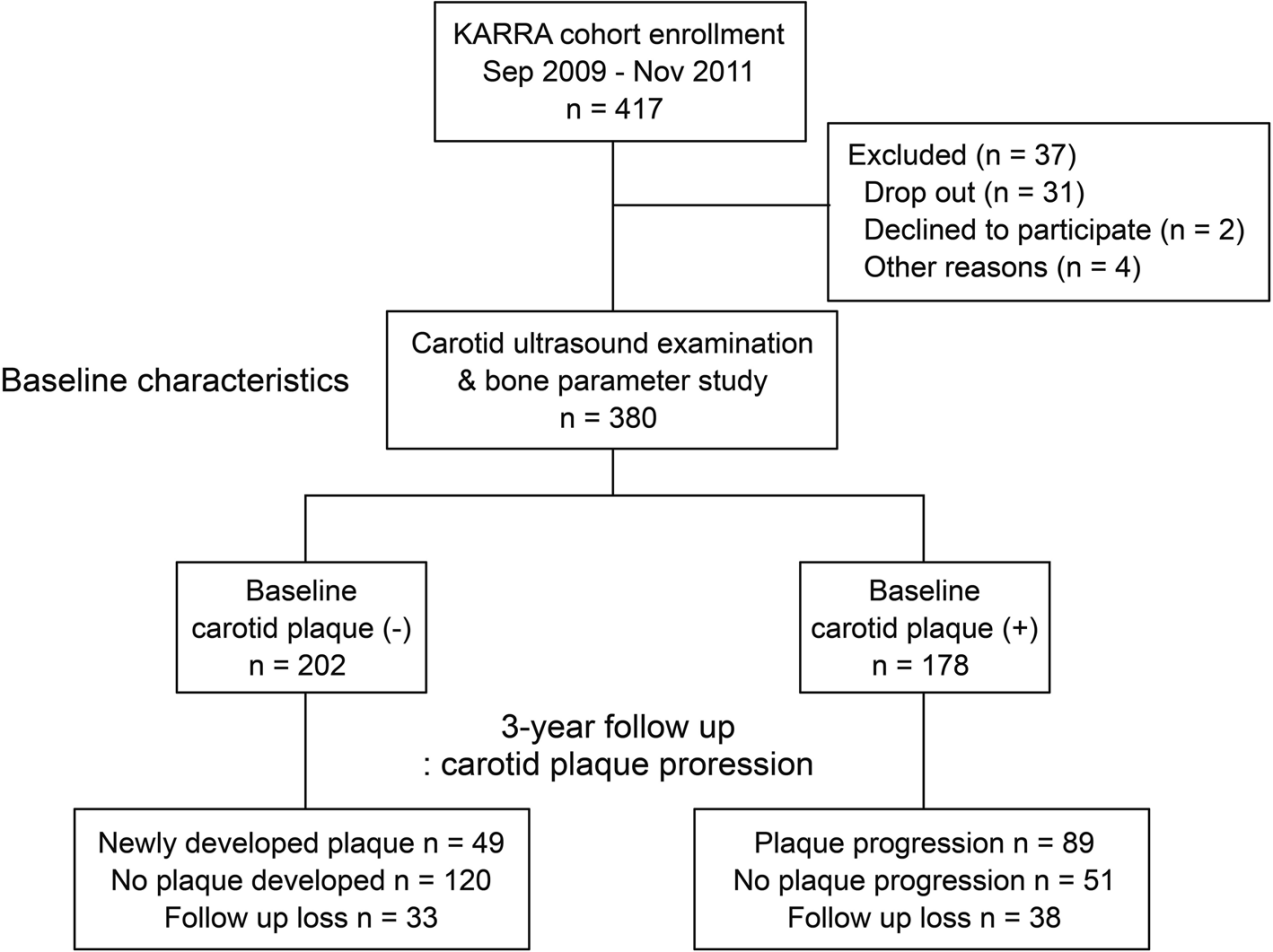
Flow chart of the KRAAR cohort study. Of the 417 RA patients recruited, we performed carotid ultrasound on 380 patients at baseline, and 3-year follow-up carotid ultrasound was performed in 309 patients. KARRA, KNUH Atherosclerosis Risk of Rheumatoid Arthritis

During the period from September 2009 to November 2011, 417 consecutive RA patients, who fulfilled the 1987 American College of Rheumatology Classification for RA, were recruited from the rheumatology clinic of KNUH to establish the KARRA cohort for a prospective, longitudinal, observation study [13]. At baseline, carotid ultrasound examinations were performed in 380 patients, 309 of whom also underwent the 3-year follow-up carotid ultrasound (Fig. 1). Ethical approval was obtained from KNUH Institutional Review Board (reference number: KNUH_09-1054), and written informed consent was obtained from all participants.

### Carotid ultrasound to assess carotid intima-media thickness (IMT) and plaque

The primary outcome in this study was the progression of carotid plaque by carotid ultrasound examination after the 3-year follow-up period. Carotid ultrasound examination was conducted using high-resolution B-mode images acquired with LOGIQ7 ultrasound system (General Electric, Milwaukee, WI) with a 10-MHz linear transducer by one qualified ultrasound technician. Carotid plaque was defined as either a focal structure that encroaches into the arterial lumen by at least 50% of the surrounding (intima-media thickness) IMT value or a thickness of > 1.5 mm as measured from the media–adventitia interface to the intima–lumen interface. The progression of carotid plaque was defined as an increase in the number of carotid plaques at the 3-year follow-up ultrasound compared to that at baseline. The carotid IMT was measured using continuous static images, 20-mm in length, obtained from 10-mm below the proximal common carotid artery through the middle of the internal carotid artery [13]. The mean and maximal IMT values were automatically analyzed with dedicated software (Intimascope TM, Media Cross, Japan).

### Demographic, clinical variables and laboratory tests

Demographic and clinical parameters of CVD- and RA-associated risk factors were obtained by experienced rheumatologists and clinical research nurses (Table 1). At baseline, body-mass index (BMI) was calculated as the weight in kilograms divided by the square of height in meters. Waist-hip ratio (WHR) was determined by dividing the waist circumference at the level of the navel by the maximum hip circumference. We diagnosed hypertension, diabetes, and hypercholesterolemia if the resting blood pressure was persistently above 140/90 mmHg, the fasting plasma glucose was ≥ 126 mg/dL or the non-fasting plasma glucose level was ≥ 200 mg/dL, and the serum low-density lipoprotein cholesterol level was ≥ 130 mg/dL, respectively, or if a person was receiving treatment for any of the above. Smoking status was classified as never-smoker, former smoker who had smoked > 100 cigarettes in their lifetime, and current-smoker. For RA-related risk factors, the disease duration was assessed by asking the date when the first symptoms of synovitis began, and a modified HAQ survey was conducted to assess the functional status in RA patients. DAS-28 with ESR (DAS28-ESR) was used to assess the activity status of RA: DAS28-ESR scores < 2.6, 2.6–3.2, 3.21–5.1, and > 5.1 were interpreted as remission, mild, moderate, and severe disease activity, respectively.

**Table 1:** Baseline demographic, clinical characteristics, and laboratory findings according to the progression of carotid plaque after 3-year follow-up of the KARRA cohort

Baseline parameters	Total cohort (*n* = 309)				No carotid plaque at baseline (*n* = 169)			
	No progression (*n* = 171)	Progression (*n* = 138)	*p*	OR (95%CI)	No plaque (*n* = 120)	New plaque (*n* = 49)	*p*	OR (95%CI)
Male (%)	21 (12.3)	33 (23.9)	0.010	2.245 (1.230–4.096)	11 (9.2)	9 (12.2)	0.546	1.383 (0.481–3.973)
Age (year)	53.06 ± 11.67	60.34 ± 9.47	< 0.001	1.065 (1.041–1.089)	48.78 ± 9.95	56.49 ± 9.09	< 0.001	1.083 (1.043–1.125)
CV risk factors								
BMI (kg/m^2^)	22.78 ± 3.58	23.14 ± 2.82	0.331	1.035 (0.966–1.109)	22.69 ± 3.73	23.02 ± 2.69	0.572	0.991 (0.957–1.026)
Waist-hip ratio	0.89 ± 0.07	0.92 ± 0.06	0.002	276.9 (7.017–10929.1)	0.88 ± 0.07	0.90 ± 0.06	0.052	154.4 (0.959–24860.9)
Hypertension	37 (21.6)	43 (31.2)	0.068	1.639 (0.982–2.736)	20 (16.7)	13 (26.5)	0.142	1.806 (0.815–4.000)
Diabetes mellitus	8 (4.7)	12 (8.7)	0.169	1.940 (0.770–4.890)	3 (2.5)	3 (6.1)	0.248	2.543 (0.495–13.063)
Hypercholesterolemia	14 (8.2)	26 (18.8)	0.006	2.603 (1.301–5.208)	9 (7.5)	8 (16.3)	0.083	2.407 (0.870–6.657)
Smoking: ever and current	21 (12.3)	38 (27.5)	0.001	2.714 (1.505–4.896)	15 (12.5)	8 (16.3)	0.510	1.366 (0.538–3.465)
Ever	12 (7.0)	19 (13.8)	0.058	2.116 (0.989–4.527)	8 (6.7)	6 (12.2)	0.233	1.953 (0.640–5.960)
Current	9 (5.3)	19 (13.8)	0.015	2.874 (1.256–6.575)	7 (5.8)	2 (4.1)	0.645	0.687 (0.138–3.429)
LDL-cholesterol (mg/dL)	109.61 ± 29.65	116.78 ± 32.69	0.046	1.007 (1.000–1.015)	105.89 ± 28.04	120.14 ± 32.55	0.005	1.016 (1.004–1.028)
Uric acid (mg/dL)	3.67 ± 1.35	4.00 ± 1.47	0.041	1.183 (1.005–1.392)	3.59 ± 1.18	3.59 ± 1.19	0.974	0.995 (0.750–1.321)
Presence of carotid plaque	51 (29.8)	89 (64.5)	< 0.001	4.274 (2.649–6.896)	NA	NA	NA	NA
Mean carotid IMT, mm	0.76 ± 0.15	0.88 ± 0.17	< 0.001	187.9 (33.2–1064.0)	0.72 ± 0.13	0.81 ± 0.12	< 0.001	256.6 (16.5–3991.9)
RA-associated risk factor								
Disease duration (year)	12.21 ± 7.89	13.74 ± 8.83	0.112	1.022 (0.995–1.051)	11.28 ± 6.28	12.23 ± 6.91	0.392	1.023 (0.972–1.076)
mHAQ	6.19 ± 7.11	7.52 ± 6.63	0.095	1.028 (0.995–1.063)	5.31 ± 5.91	7.43 ± 6.74	0.044	1.053 (1.000–1.109)
DAS28-ESR	3.21 ± 1.12	3.41 ± 1.26	0.123	1.162 (0.960–1.406)	3.18 ± 1.13	3.26 ± 1.11	0.676	1.065 (0.793–1.432)
ESR (mm/h)	23.91 ± 20.08	28.39 ± 21.28	0.059	1.011 (0.999–1.022)	23.81 ± 20.80	25.75 ± 17.77	0.570	1.005 (0.988–1.021)
CRP (mg/dL)	0.39 ± 0.68	0.53 ± 1.14	0.177	1.201 (0.906–1.593)	0.41 ± 0.76	0.41 ± 0.64	0.983	1.005 (0.634–1.593)
Rheumatoid factor (IU/mL)	61.03 ± 80.77	90.22 ± 131.96	0.025	1.003 (1.000–1.005)	56.76 ± 55.17	67.85 ± 109.67	0.347	1.002 (0.998–1.006)
ACPA (Unit/mL)	285.34 ± 217.48	296.17 ± 234.73	0.699	1.000 (0.999–1.001)	290.94 ± 218.68	267.48 ± 224.36	0.565	1.000 (0.998–1.001)
Steroid ≥ 5 mg	20 (11.9)	13 (9.4)	0.486	0.770 (0.368–1.609)	13 (10.9)	4 (8.2)	0.509	0.725 (0.224–2.344)
Methotrexate dosage (mg/week)	10.77 ± 3.87	11.28 ± 3.31	0.242	1.040 (0.973–1.111)	10.85 ± 3.9	11.06 ± 3.08	0.751	1.016 (0.923–1.118)
TNF inhibitors	10 (5.8)	9 (6.5)	0.806	1.123 (0.443–2.846)	6 (5.0)	2 (4.1)	0.799	0.809 (0.157–4.151)
Bone parameter								
Osteocalcin (ng/mL)	7.17 ± 3.29	7.71 ± 3.55	0.170	1.048 (0.979–1.122)	7.12 ± 2.70	8.39 ± 4.09	0.021	1.126 (1.014–1.251)
Serum CTX-I (pg/mL)	0.37 ± 0.19	0.42 ± 0.24	0.014	3.972 (1.288-12.253)	0.36 ± 0.18	0.44 ± 0.22	0.014	8.171 (1.446–46.154)
BMD mean (g/cm^2^)								
Lumbar BMD	1.06 ± 0.19	1.03 ± 0.21	0.187	0.436 (0.127–1.496)	1.07 ± 0.17	1.03 ± 0.20	0.154	0.207 (0.024–1.818)
Femur BMD	0.86 ± 0.14	0.82 ± 0.16	0.007	0.106 (0.020–0.554)	0.87 ± 0.13	0.82 ± 0.14	0.026	0.045 (0.003–0.719)
Radius BMD	0.60 ± 0.12	0.55 ± 0.14	0.004	0.059 (0.008–0.421)	0.61 ± 0.11	0.56 ± 0.13	0.010	0.019 (0.001–0.426)
Osteopenia or osteoporosis (T-score < − 1.0)								
Lumbar	71 (45.5)	73 (60.8)	0.015	1.859 (1.147–3.015)	48 (43.6)	25 (61.0)	0.058	2.018 (0.971–4.196)
Femur	67 (39.2)	39 (50.0)	0.065	1.552 (0.986–2.443)	40 (36.7)	24 (58.5)	0.016	2.435 (1.170–5.070)
Radius	70 (45.5)	80 (69.6)	< 0.001	2.743 (1.650–4.560)	41 (37.6)	24 (60.0)	0.015	2.488 (1.185–5.224)

Abbreviations: *CV* cardiovascular; *BMI* body mass index; *LDL* low-density lipoprotein; *mHAQ* Modified Health assessment questionnaire; *DAS28* Disease Activity Score 28; *ESR* erythrocyte sedimentation rate; *CRP* C-reactive protein; *ACPA* anti-citrullinated protein antibody; *TNF* tumor necrosis factor; *CTX-I* C-terminal telopeptides type I collagen; *BMD* bone mineral density; *OR* odds ratio; *CI* confidence interval; *NA* not available

Morning venipuncture blood samples were obtained after a 12-h fast and were analyzed in KNUH clinical laboratories. Blood tests for metabolic diseases (fasting glucose, lipid profile, insulin resistance, and serum uric acid) and RA (ESR, CRP, rheumatoid factor and anti-citrullinated peptide antibody (ACPA)) were checked. Serum osteocalcin and C-terminal telopeptide of type-I collagen (CTX-I) was determined by automated radioimmunoassay and the electro-chemiluminescence method, respectively. BMD was evaluated at the lumbar spine, femoral neck, and distal radius by dual-energy X-ray absorptiometry using a Discovery W densitometer (Hologic®, Marlborough, MA). BMD values were expressed in absolute values (g/cm^2^) and *T* scores, which defines scores between − 1 and − 2.5 as osteopenia and scores below − 2.5 as osteoporosis.

### Statistical analyses

The association with the primary end point of carotid plaque progression was compared using a Student *t* test for continuous variables and with the chi-square test for discrete parameters. The odds ratio (OR) for continuous variables was assessed by logistic regression with a single continuous variable.

Multivariable analyses were performed in 4 models by binary logistic regression analysis, with forward selection of the variables that demonstrated a statistical significance of *p* value < 0.1 in univariate analyses. The first model included only clinical variables as the covariate, such as age, male sex, WHR, hypertension, hypercholesterolemia, smoking status, serum LDL-cholesterol, uric acid level, presence of baseline carotid plaque, mHAQ, RF, and ESR. The second and third model added the bone parameters of serum CTX-1 and distal radius BMD, respectively, and the fourth model included both bone parameters to clinical variables. The goodness of fit of each model was determined with the Nagelkerke *R*^2^ coefficient of determination. All statistical analyses were performed with the statistical package software IBM SPSS® for Windows version 25.

## Results

### Progression of carotid plaque in 3-year interval and its risk factors

The 3-year follow-up carotid ultrasound performed on these 309 RA patients revealed the development of new plaque in 49 of the 169 patients without baseline carotid plaque, and the progression of plaque in 89 of the 140 patients with baseline plaque. Consequently, a total of 138 patients (44.7%) demonstrated carotid plaque progression, but the other 171 patients did not (Fig. 1).

When we compared the baseline demographic and clinical characteristics of the total cohort, the following factors were significantly associated with the progression of carotid plaque in the univariate models: male sex, age, WHR, hypercholesterolemia, smoking, LDL-cholesterol, and serum uric acid level. In addition, the progression of carotid plaque in 3-year was significantly high in the patients with carotid plaque (*p* < 0.001) and thick carotid IMT (*p* < 0.001) at baseline. Among the RA-related factors, only the level of rheumatoid factor was associated with the progression of carotid plaque after 3 years (*p* = 0.025), although the current ESR was marginally associated (*p* = 0.059). On the other hand, the parameters for disease activity (DAS-28 ESR), disease duration, or medications, including steroid, MTX, or biologics, failed to predict the progression of carotid plaque at the 3-year follow-up (Table 1, Supplementary Table 1, Supplementary Figure 2). Among bone-related factors, the patients with a higher serum CTX-I level (*p* = 0.014), and a lower femur and radius BMD (*p* = 0.007 and 0.004, respectively) had a higher incidence of carotid plaque progression (Table 1, Supplementary Figure 1).

We then conducted a subgroup analysis on the 169 RA patients who did not have carotid plaque at baseline. The development of new carotid plaque was common in elderly patients (*p* < 0.001) and in those with higher LDL-cholesterol (*p* = 0.005) and mean carotid IMT at baseline (*p* < 0.001). There was no significant association between the RA-associated risk factors and the development of new carotid plaque. The patients with an increased level of serum osteocalcin (*p* = 0.021) and CTX-1 (*p* = 0.014) at baseline had a higher incidence of carotid plaque development at the 3-year follow-up. In addition, lower baseline femur and radius BMDs were also significant risk factors for the development of carotid plaque at the 3-year follow-up (p = 0.016 and 0.010, respectively) (Table 1, Supplementary Figure 1).

### Parameters reflecting bone turnover as an independent predictor of subclinical atherosclerosis

To identify the independent baseline risk factor for carotid plaque progression, variables with statistical significance of *p* < 0.10 in univariate analyses were subjected to backward stepwise multiple logistic regression analysis. When the variables associated with traditional CVD risk factors and RA-related factors were analyzed in model 1, the progression of carotid plaque was independently associated with the patient’s age, hypercholesterolemia, smoking, and the presence of carotid plaque at baseline. These 4 variables explained 24.3% of the variance of this model (R^2^) for carotid plaque progression. Then, we added the bone parameters, CTX-I and distal radius BMD, to clinical models 2 and 3, respectively. The serum CTX-I and distal radius BMD remained as independent risk factor and were the most important predictors of carotid plaque progression among those evaluated; the β coefficient of CTX-1 and radius BMD were 1.539 and -3.057, respectively. These parameters could increase the explained variance of the model (*R*^2^) to 27.3% and 25.6%, respectively. Adding both CTX-I and distal radius BMD to the clinical risk factors in model 4 could explain 29.7% of the variance in the regression model for predicting carotid plaque progression (Table 2).

**Table 2:** Regression coefficients and OR (95% CI) of best models based on backwards regression analysis for the progression of carotid plaque over 3 years in RA patients

	Model 1: Clinical factors (*n* = 252)		Model 2: CTX-I (*n* = 244)		Model 3: BMD radius (*n* = 223)		Model 4: CTX-I+ BMD radius (*n* = 216)	
Baseline parameters^a^	*β* coefficient (SE)	OR (95% CI)	*β* coefficient (SE)	OR (95% CI)	*β* coefficient (SE)	OR (95% CI)	*β* coefficient (SE)	OR (95% CI)
Age	0.036 (0.014)	1.037 (1.010–1.065)^**^	0.040 (0.014)	1.041 (1.012–1.070)^**^	–	–	–	–
Hypercholesterolemia	0.896 (0.381)	2.450 (1.162–5.164)^*^	1.024 (0.393)	2.784 (1.290–6.010)^**^	0.963 (0.390)	2.621 (1.221–5.624)^*^	1.099 (0.404)	3.002 (1.360–6.626)^**^
Smoking: ever and current	0.611 (0.212)	1.843 (1.217–2.791)^**^	0.649 (0.216)	1.913 (1.252–2.924)^*^	0.903 (0.261)	2.467 (1.480–4.111)^**^	0.988 (0.272)	2.687 (1.577–4.579)^***^
Presence of carotid plaque	0.992 (0.288)	2.697 (1.534–4.739)^**^	1.002 (0.295)	2.723 (1.527–4.853)^**^	1.291 (0.282)	3.637 (2.095–6.316)^***^	1.353 (0.291)	3.869 (2.190–6.838)^***^
Serum CTX-I (pg/mL)	NA	NA	1.539 (0.667)	4.662 (1.262–17.219)	NA	NA	1.718 (0.731)	5.574 (1.331–23.341)^*^
BMD radius (g/cm^2^)	NA	NA	NA	NA	–3.057 (1.193)	0.047 (0.005–0.488)^*^	–3.667 (1.250)	0.026 (0.002–0.296)^**^
*R*^2^ (%)	24.3		27.3		25.6		29.7	

^a^Excluded variables which has statistical significance of *p* < 0.10 for the progression of carotid plaque in univariate analysis were as follows: sex, waist-hip ratio, the presence of hypertension, serum LDL-cholesterol level, serum uric acid level, mHAQ, RF, and ESR

^*^p < 0.05; ^**^p < 0.01; ^***^p < 0.001. *R*^2^: Nagelkerke *R* square

Abbreviations: *β* partial regression coefficient; *SE* standard error; *OR* odds ratio; *CI* confidence interval; *CTX-I* C-terminal telopeptides type I collagen; *BMD* bone mineral density; *NA* not-available; *R*^2^ coefficient of determination

### Synergistic interactions between serum CTX-I and radius BMD in subclinical atherosclerosis

**Fig. 2:**
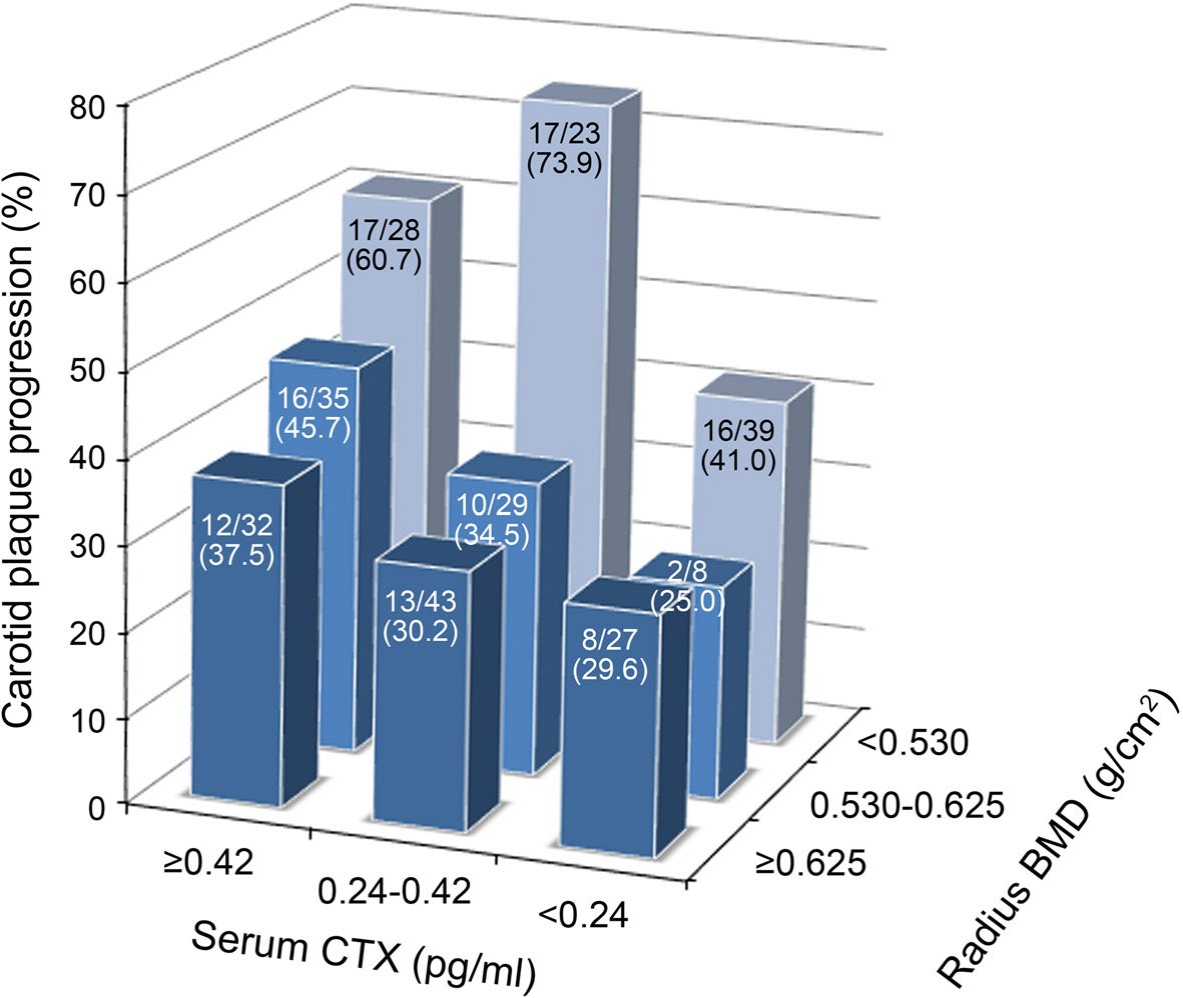
Three-dimensional bar graph showing the interaction of the serum CTX-I level and radius BMD on carotid plaque progression

The incidence of plaque progression was compared according to the serum CTX-I and radius BMD score to clarify the interaction between these two variables. Patients in the upper tertile of radius BMD (≥ 0.625 g/cm^2^) showed only about 30% of the plaque progression incidence as the middle and lower tertile groups of serum CTX-I, and it increased to 37.5% in the upper CTX-I tertile group. In the middle tertile group of radius BMD (0.530–0.625 g/cm^2^), the incidence of carotid plaque progression was gradually increased by the serum CTX-I level, that is 25.0%, 34.5% and 45.7% in the lower, middle, and upper CTX-I tertile groups, respectively. In the lower tertile group of radius BMD (< 0.530 g/cm^2^), plaque progression was observed in 42% of the patients in the lower tertile of serum CTX-I, and in nearly 60%–70% of the patient in middle and upper tertiles (Fig. 2).

## Discussion

We investigated the association of clinical, RA-related, and bone parameters for the progression of atherosclerosis by measuring the number of carotid plaques over 3-year interval in a 380-patient RA cohort. This study identifies a prospective and longitudinal relationship between the progression of carotid atherosclerosis and lower distal radius BMD and higher serum CTX-I levels at baseline, which reflects bone resorption and osteoclast activity.

A number of epidemiologic studies have reported an association between subclinical atherosclerosis and BMD, not only in general population, but also in patients with inflammatory disease [14–17]. A Norwegian population-based study found a twofold increase in echogenic carotid plaque in subjects in the highest quartile of distal forearm BMD compared to those in the lowest quartile, while the presence of echolucent plaque was not associated with distal forearm BMD [14]. A lupus case-control study reported a twofold increase in echolucent carotid plaque and a fourfold increase in echogenic plaque in patients with a lower hip BMD T-score of <− 1.0 standard deviation compared to controls [15]. Furthermore, the lupus patients in the lowest and middle tertiles of hip BMD were found to have about a threefold increase in the carotid plaque index compared to the patients in the highest tertile of hip BMD, but the mean carotid IMT did not differ among the hip BMD tertiles [16]. More recently, a study of patients with psoriatic arthritis found an association between the presence of carotid plaque and total vertebral BMD and suggested a link between interleukin-33 and its decoy receptor, soluble ST2, as a pathophysiologic mediator [17]. Overall, the studies to date have confirmed that a lower BMD is associated with the carotid plaque burden as a surrogate for subclinical atherosclerosis.

Another interesting finding of this study was that carotid plaque progressed faster in patients with a higher baseline CTX-1 level. To our knowledge, this is the first study to demonstrate the connection between atherosclerosis and CTX-1, the serum marker for osteoclast activity. There are two kinds of carboxy-terminal cross-linked telopeptide of type-I collagen: ICTP and CTX-1. Type-I collagen is degraded by the matrix metalloproteinase-2, metalloproteinase-9, metalloproteinase-13, or metalloproteinase-14, which produces ICTP, and then cathepsin K degrades the ICTP epitope, which produces CTX-1 [18]. As cathepsin K is produced mainly by activated macrophages and osteoclasts, the serum level of CTX-1 reflects the activity of macrophages and osteoclasts [19]. Actually, an atherosclerotic plaque lesion has foam cells that originate from a macrophage lineage, which can also produce cathepsin K, and it can increase the levels of CTX-1 through degradation of the atherosclerotic plaque [19, 20]. However, it seems unlikely that this local proteolytic process can affect the serum CTX-1 level, considering the bone mass of the human body [20]. In our data, the upper tertile group of the distal radius BMD was not affected by the baseline serum CTX-1 level, showing only 30% of the progression of carotid plaque over the 3-year period. On the other hand, plaque progression in the middle and lower tertile groups was significantly affected by the baseline serum CTX-1 level (Fig. 1). This suggests that serum CTX-1 is closely connected to bone metabolism, rather than plaque remodeling itself.

The mechanisms connecting the atherosclerotic burden and enhanced bone metabolism are still poorly understood, but there are several potential hypotheses. The main effector cells in atherosclerosis and bone metabolism are foam cells and osteoclasts, respectively, and these two cells commonly originate from the monocyte-macrophage lineage [21]. Inflammatory cytokines, such as tumor necrosis factor-α and interleukin-6, can enhance the differentiation of the macrophage and are responsible for the interaction between bone loss and atherosclerosis in RA patients [22]. Another possibility is the presence of a common mediator between atherosclerosis and osteoporosis, such as osteoprotegerin (OPG) or omentin-1. As a decoy receptor for RANKL, OPG can be elevated in the osteoporosis patient [23, 24], and this increased OPG can suppress apoptosis of endothelial and vascular smooth muscle cells as a decoy receptor for TRAIL, leading to atherosclerosis [25–27]. In addition, omentin-1, a novel visceral adipose tissue-derived cytokine, is known to attenuate both bone loss and the progression of atherosclerosis [28, 29]. Finally, increased bone metabolism itself can be responsible for the association between osteoporosis and atherosclerosis. Evidence from bisphosphonate studies has shown that the inhibition of osteoclast function with bisphosphonate has an anti-atherosclerotic effect in both the normal population and lupus patients [30, 31]. Considering bisphosphonate accumulates mainly in bone by binding to hydroxyapatite crystals [32], the decreased bone metabolism by bisphosphonate and the consequent reduction of calcium-phosphate efflux or serum osteocalcin, an osteoblast-derived bone Gla (γ-carboxyglutamic acid-containing) protein, can attenuate the progression of atherosclerosis [33, 34]. These hypothesis explaining the connection between osteoclast activation and atherosclerosis can be commonly applied in inflammatory diseases other than RA, and actually evidences have shown the enhanced atherosclerosis in many autoimune diseases. Further investigation is needed to determine whether osteoclast activation in inflammatory condition has a pathogenic role in the progress of atherosclerosis or if it is a just epiphenomenon of enhanced inflammation.

Another potential mechanism for explaining the link between atherosclerosis and osteoporosis is bone anabolic pathways such as canonical Wnt and parathyroid hormone (PTH) signaling [35]. The canonical Wnt signaling is well known to enhance bone formation through the differentiation of precursors into osteoblast [36]. Recent evidences have shown that Wnt signaling inhibits foam cell formation by inhibiting intracellular lipid accumulation, which consequently suppresses the development of atherosclerosis [37]. Interestingly, RA patients have higher level of serum Dickkopf-1, the endogenous inhibitor of Wnt signaling, compared to controls, and it correlates well with RA disease activity and more severe bone destruction [38, 39]. PTH also has been reported to be associated with atherosclerosis and cardiovascular diseases [40, 41]. The serum level of PTH in RA patients was 40% higher than in controls [42], and it was associated with bone erosion in RA [43]. The attenuation of canonical Wnt signaling and enhanced PTH signaling can explain the correlation between osteoporosis and atherosclerosis in RA patients.

Contrary to our expectation, the disease activity of RA at baseline assessed by DAS28-ESR did not affect the progression of carotid plaque as well as the mean cIMT (Table 1, supplementary figure 3 and supplementary table 1). The 3-year prospective GIRRCS study on 841 Italian RA patients revealed that patients in the remission state of DAS28-ESR (< 2.6) at baseline had a significantly lower subclinical atherosclerotic lesion on their carotid or peripheral artery compared to active RA patients after 3 years [44]. Given our contrasting findings, this negative correlation of disease activity with atherosclerosis progression may result from the well-controlled RA activity in our study population. In fact, the mean baseline DAS28-ESR in our study was 3.2 and 3.4 in plaque non-progression and progression groups, respectively. A prospective, observational study showed if the RA activity is well-controlled to remission or low activity (DAS28-ESR ≤ 3.2) during a 3-year follow-up period, the risk of atherosclerosis progression is even similar to a non-RA control group [45]. Although our negative result may derive from the weak power to detect a small difference in this study, the RA activity does not seem to have a significant effect on the progression of atherosclerosis in patients whose RA disease activity is well-controlled.

## Conclusions

We investigated the potential risk for the progression of carotid plaque in RA patient in this prospective, observational study. The bone parameters of lower distal radius BMD and elevated serum CTX-1 level, along with hyperlipidemia, smoking, and the presence of carotid plaque at baseline were independent risk factors for the progression of atherosclerosis in RA patients. Our study sheds light on the connection between bone metabolism and atherosclerosis. Further clinical investigation is needed to determine whether this connection is RA-specific or a common phenomenon in the general population.

## Supplementary Information

**Additional file 1: **Supplementary **Figure S1**. Forest plot showing the odds ratio for risk of the progression of carotid plaque. Supplementary **Figure S2**. RA disease activity at baseline and changes in mean carotid IMT. Supplementary **Table S1**. Disease activity and the progression of carotid plaque in RA patients

## Data Availability

The data that support the findings of this study are available from the corresponding author, YM Kang, upon reasonable request.
