# Supplementary material for: Radial BMD and serum CTX-I can predict the progression of carotid plaque in rheumatoid arthritis: a 3-year prospective cohort study
Source: Arthritis Res Ther. 2021 Oct 13;23:258. doi: 10.1186/s13075-021-02642-4 (PMC8513174; doi:10.1186/s13075-021-02642-4)
Supplement: Supplementary file 1 — Additional file 1: Supplementary Figure S1. Forest plot showing the odds ratio for risk of the progression of carotid plaque. Supplementary Figure S2. RA disease activity at baseline and changes in mean carotid IMT. Supplementary Table S1. Disease activity and the progression of carotid plaque in RA patients [file 13075_2021_2642_MOESM1_ESM.docx]

**Baseline radial bone mineral density and serum C-terminal telopeptide of type-I collagen independently predict the progression of carotid plaque in rheumatoid arthritis: a three-year prospective cohort study**

Seungwoo Han^1^, Na-Ri Kim^1^, Jong-Wan Kang^2^, Jung-Su Eun^1^, and Young-Mo Kang^1^

^1^Division of Rheumatology, Department of Internal Medicine, Kyungpook National University, School of Medicine, Daegu, Republic of Korea.

^2^Division of Rheumatology, Department of Internal medicine, Daegu Fatima Hospital, Daegu, Republic of Korea

Running title: Bone parameters and carotid plaque in RA

**Supplementary data**

Supplementary figure 1. Forest plot showing the odds ratio for risk of the progression of carotid plaque.


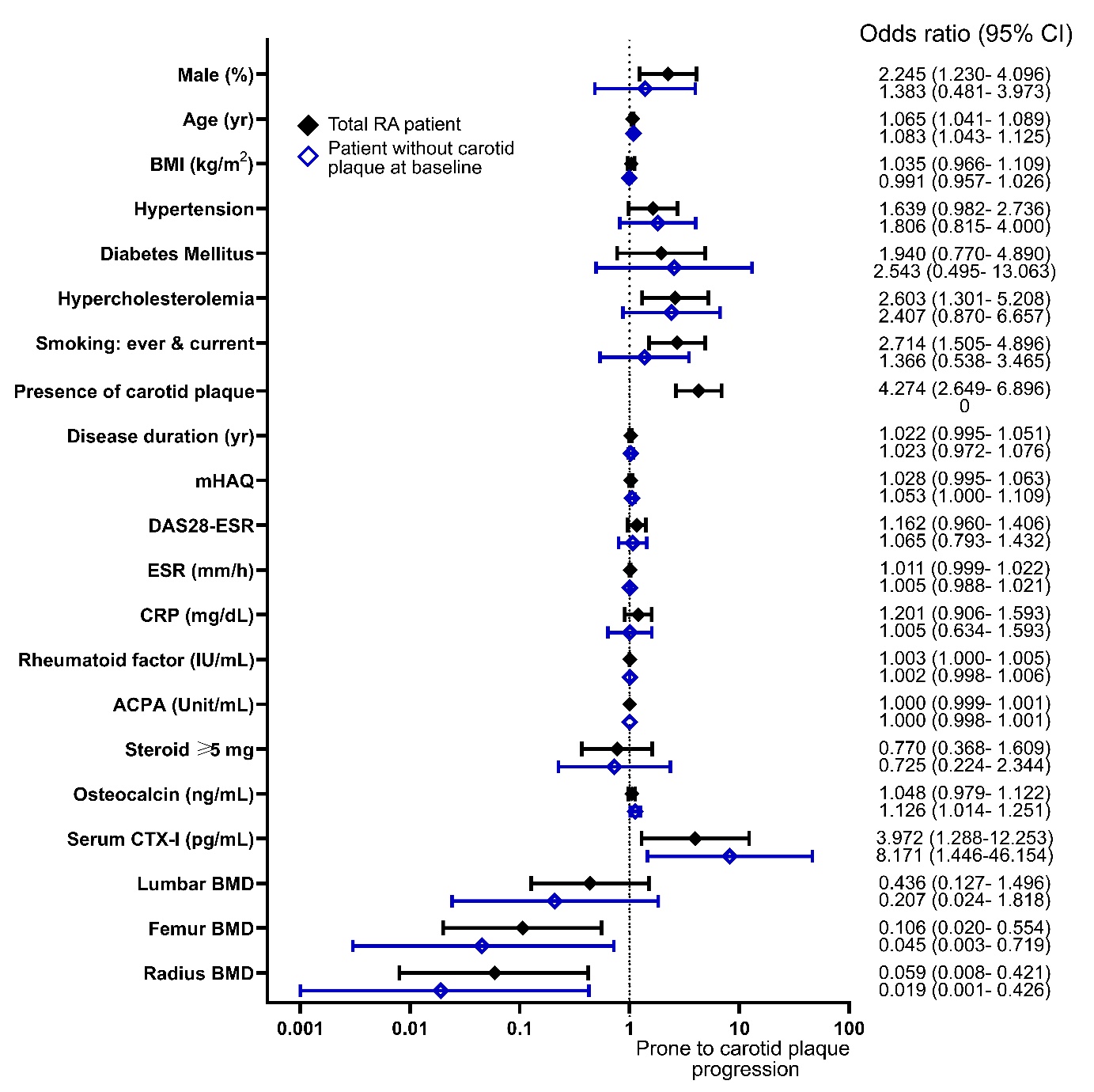


Supplementary figure 2. RA disease activity at baseline and changes in mean carotid IMT


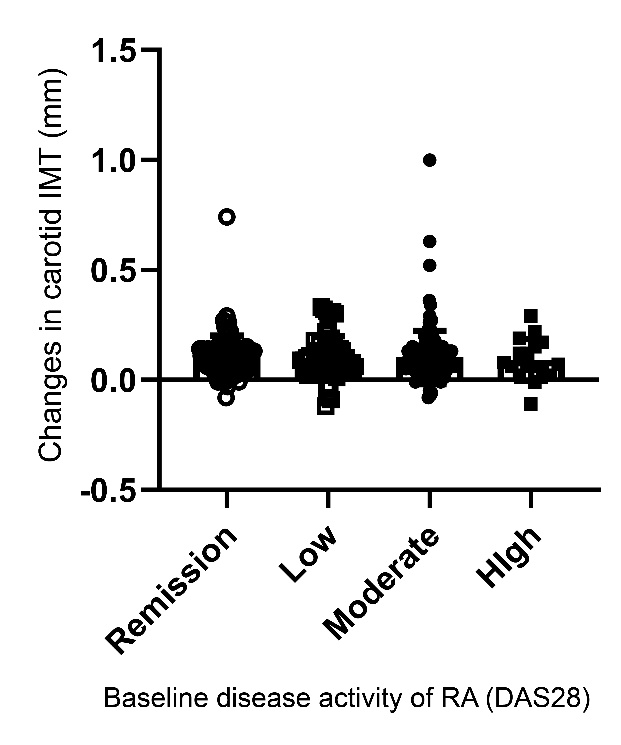


Supplementary Table 1. Disease activity and the progression of carotid plaque in RA patients

| DAS28-ESR | Remission (<2.6) | Low  (2.6–3.2) | Moderate  (3.21–5.1) | High (> 5.1) | Total |
| --- | --- | --- | --- | --- | --- |
| Aggravation of carotid plaque | 37/90 (41.1) | 29/63 (46.0) | 61/138 (44.2) | 10/17 (58.8) | 137/308 |
| Newly developed carotid plaque | 16/58 (27.6) | 11/31 (35.5) | 19/73 (26.0) | 3/7 (42.9) | 48/169 |
